# Pharmacogenomics of Allopurinol and Sulfamethoxazole/Trimethoprim: Case Series and Review of the Literature

**DOI:** 10.3390/jpm11020071

**Published:** 2021-01-26

**Authors:** Ogechi Ikediobi, Jeremy A. Schneider

**Affiliations:** Department of Dermatology, University of California, San Diego, CA 92093, USA; j3schneider@health.ucsd.edu

**Keywords:** pharmacogenomics, dermatology, drug-induced hypersensitivity syndrome, Stevens–Johnson syndrome, toxic epidermal necrolysis, severe cutaneous adverse drug reaction

## Abstract

Severe cutaneous adverse drug reactions (SCAR) such as the Stevens–Johnson syndrome/toxic epidermal necrolysis (SJS/TEN) and drug rash with eosinophilia and systemic symptoms/drug-induced hypersensitivity syndrome (DIHS) can be induced by a plethora of medications. The field of pharmacogenomics aims to prevent severe adverse drug reactions by using our knowledge of the inherited or acquired genetic risk of drug metabolizing enzymes, drug targets, or the human leukocyte antigen (HLA) genotype. Dermatologists are experts in the diagnosis and management of severe cutaneous adverse drug reactions (SCAR) in both the inpatient and outpatient setting. However, most dermatologists in the US have not focused on the prevention of SCAR. Therefore, this paper presents a case series and review of the literature highlighting salient examples of how dermatologists can apply pharmacogenomics in the diagnosis and especially in the prevention of SCAR induced by allopurinol and sulfamethoxazole/trimethoprim, two commonly prescribed medications.

## 1. Introduction

Severe cutaneous adverse drug reactions (SCAR) such as the epidermal necrolysis characteristic of the Stevens–Johnson syndrome (SJS)/toxic epidermal necrolysis (TEN) and the morbilliform exanthematous rash with the systemic involvement characteristic of drug-induced hypersensitivity syndrome (DIHS) are classified as delayed type (type-IV) hypersensitivity reactions [1]. Type IV hypersensitivity reactions are characterized by the activation of CD4 and CD8 lymphocytes and often develop 7 days to several weeks after drug exposure [1]. Many studies have elucidated an interaction between human leukocyte antigen (HLA) and drugs in inducing T-cell receptor (TCR) signaling and subsequent lymphocyte activation. There are several examples of specific HLA alleles associated with SCAR. Notably, carbamazepine and lamotrigine, commonly used anticonvulsant drugs, are associated with SCAR in patients of Asian ethnicity and the HLA-B*15:02 allele and in patients of European ancestry and the HLA-A*31:01 allele [1]. Similarly, the presence of the HLA-B*57:01 allele is associated with abacavir DIHS [1]. In all these examples, pre-emptive screening has been shown to reduce the incidence of SCAR [1].

Allopurinol-induced SCAR is associated with the presence of the HLA-B*58:01 allele [1]. Pre-emptive screening for the HLA-B*58:01 allele has been shown to reduce the incidence of SCAR in new users of allopurinol in Europe, Asia and the US [2]. However, screening for the HLA-B*5801 allele is not as widespread in the US compared to Taiwan and other Asian countries [1,2].

There are two main reasons for the difference in the clinical implementation of pre-emptive screening between Asian countries and the US. First, Taiwan and China have a relatively homogeneous population with very little admixture, therefore genetic screening is much easier. Second, the reimbursement of testing is supported by national health insurance in Taiwan, China and Korea [1]. In the US, Medicare does reimburse for pharmacogenetic testing. However, there are no transparent policies with regards to pharmacogenetic testing from other health insurance agencies. However, the cost of testing nowadays is negligible and can often be justified with supporting medical literature from medical journals or the FDA drug label [1]. In the case of allopurinol, although there is no FDA label warning of the association between SCAR and the presence of HLA-B*58:01, the American College of Rheumatology (ACR) guidelines recommend pre-emptive testing in select Asian populations.

Inherited differences in the metabolism of drugs have also been shown to be associated with the development of SCAR. N-acetyl transferase 2 (NAT2) is an enzyme expressed in the human liver and intestines that acetylates arylamine carcinogens and many medications including: dapsone, isoniazid, hydralazine and sulfonamides [3]. The NAT2 slow acetylator phenotype is associated with isoniazid-induced hepatitis, sulfamethoxazole-induced cutaneous hypersensitivity and the increased frequency of certain cancers [3,4]. The US FDA label for sulfamethoxazole states that patients with a slow acetylator status are at increased risk of hypersensitivity reactions. In spite of this knowledge, it is not common practice to perform pre-emptive screening for NAT2 acetylator status prior to treatment with isoniazid or sulfamethoxazole. In fact, to our knowledge, there are no published studies demonstrating the implementation of NAT2 genetic testing to diagnose sulfamethoxazole-induced SCAR or pre-emptive NAT2 genetic testing to prevent sulfamethoxazole-induced SCAR.

## 2. Patients and Methods

Here, we present a case series at a single US institution describing the clinical management of SCAR induced by allopurinol and sulfamethoxazole/trimethoprim (Bactrim), emphasizing the role of inherited genetic risk and how dermatologists can play a larger role in preventing SCAR. The cases were treated at the University of California, San Diego (UCSD) in 2016 and 2020, respectively. The HLA-B*58:01 genetic test was performed at the UCSD Center for Advanced Laboratory Medicine (San Diego, CA, USA). The N-acetyltransferase 2 (*NAT2*) genetic test was performed at the Mayo Clinic Laboratory (Rochester, MN, USA). Informed consent was obtained from patients for the *NAT2* genetic test as well as for the publication of the accompanying images.

## 3. Case 1: Allopurinol Drug-Induced Hypersensitivity Syndrome with Toxic Epidermal Necrolysis-Like Phenotype

**Figure 1:**
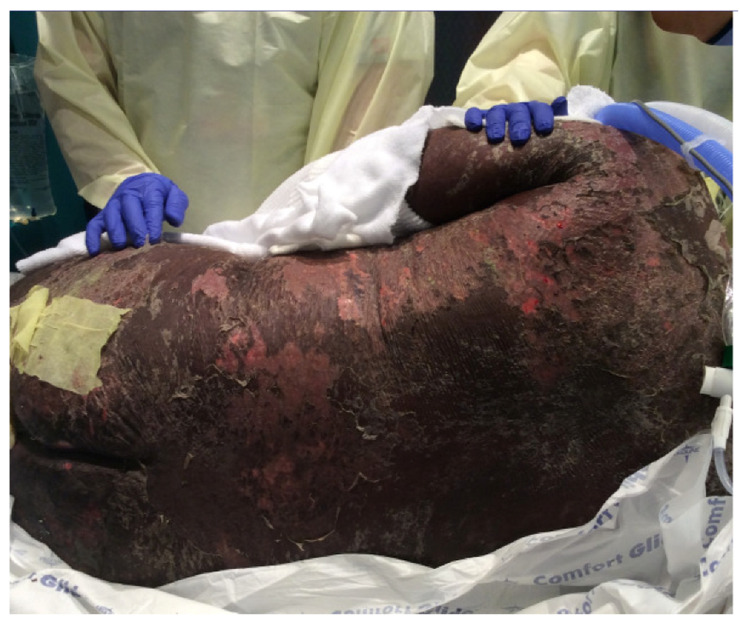
Patient with allopurinol drug-induced hypersensitivity syndrome (DIHS). Erythroderma and epidermal necrolysis with over 80% body surface area (BSA) involvement.

Mr. J, a 42-year-old man with a past medical history of type 2 diabetes mellitus, hypertension and gout presented to the Emergency Department in August 2016 with a three-day history of facial swelling and a morbilliform rash on his face and torso. He was initially diagnosed as having an allergic reaction and was prescribed antihistamines and prednisone and sent home. He presented a few days later with a notable progression of the rash that developed into bullae on his face, neck, trunk, extremities and involved his oral and genital mucosa. His serum chemistries were notable for an elevated creatinine 2.04 mg/dL (baseline creatinine 2.06 mg/dL) and elevated liver enzymes AST 135 units/L and ALT 270 units/L. There was no eosinophilia. On review of his medication history, he had been prescribed allopurinol, 300 mg daily, eight weeks prior for the management of gout. Given the severity of his rash and concern for DIHS, an HLA-B*58:01 test was ordered and he was positive for the risk allele (Table 1). Over the next week, his morbilliform exanthem progressed, taking on a dusky appearance, followed by extensive denudation; clinical history and repeat histologic sampling confirmed a diagnosis of DIHS with a TEN-like phenotype (sometimes classified as an “overlap” syndrome). He was transferred to the Burn ICU as per protocol and received treatment with Intravenous immunoglobulin (IVIG) IVIG and methylprednisolone. By October 2016, he developed septic shock and was treated appropriately with antibiotics. However, given the 80% body surface area (BSA) involvement of the epidermal necrosis (Figure 1) as well as his persistently elevated troponins, he developed unstable supraventricular tachycardia and died.

In 2016, the American College of Rheumatology (ACR) guidelines for the management of gout recommended the HLA-B*58:01 test for patients of Han Chinese, Thai or Korean descent. That recommendation was based on the initial studies that elucidated the relationship between HLA-B*58:01 and allopurinol hypersensitivity syndrome which were performed in Asia [2]. In 2017, a large cohort study of US patients initiated on allopurinol showed that among all ethnicities represented in the cohort, Native Hawaiian/Pacific Islanders, Asians and African Americans/Blacks had a higher risk of SCARs compared with Caucasians and Hispanics [5]. The findings of the study reflect the now known population frequency of HLA-B*58:01: 5.8–22% in Pacific Islanders, 7–20% in Asians, 2.6–6.4% of US Blacks, 1% in Hispanics/Mexicans and 1–2.9% in Caucasians [5]. A strength of this US-based study was the elucidation of other associated risk factors for allopurinol DIHS: female gender, age over 60 years, baseline chronic kidney disease (CKD) and an initial allopurinol dose greater than 100 mg daily [5]. Mr. J, in addition to being positive for the HLA-B*58:01 allele, had baseline CKD and was initiated on an allopurinol dose greater than 100 mg daily. All those factors combined put him at an increased risk of experiencing allopurinol DIHS but because he was not of Asian ethnicity, per 2012 ACR guidelines, he was not considered at high risk.

A cost-effectiveness analysis of HLA-B*58:01 screening to guide allopurinol use in the treatment of gout found that HLA-B*58:01 testing was cost-effective for African Americans and Asians but not Caucasians and Hispanics [6]. However, the study also found that compared to no testing, universal testing cost more and was more effective for all ethnicities [6]. This is because regardless of ethnicity, if a patient carries the HLA-B*58:01 allele and has other risk factors such as CKD or age over 60 years, they are at increased risk of allopurinol DIHS [2].

In light of the recent findings, the revised 2020 ACR guidelines now recommend testing for HLA-B*58:01 in patients of South East Asian ancestry (e.g., Han Chinese, Thai or Korean descent) and African Americans [7]. The 2020 ACR guidelines also conditionally recommend against universal HLA-B*58:01 testing [7].

## 4. Case 2: Allopurinol Drug-Induced Hypersensitivity Syndrome in a Hispanic Patient

Ms. M, an 81-year-old woman with a past medical history of CKD, hypertension and hyperlipidemia was transferred to UCSD hospital in March 2020 with a 2-week history of a morbilliform rash and worsening kidney function. The rash was preceded by prodrome of fever T100.4F and malaise. Two days after rash onset, the patient presented to her nephrologist who noted that her serum creatinine was elevated at 3.62 mg/dL (baseline 1.38 mg/dL). Her urinalysis was notable for proteinuria and hematuria. Upon review of her medication history, it was noted that the only recent change was that the patient was prescribed allopurinol 100 mg daily for hyperuricemia 5 weeks prior to the rash onset. Allopurinol was discontinued and the patient was prescribed a 4-day course of oral prednisone. Her rash improved while on prednisone, however, upon the discontinuation of prednisone, the rash recurred with an associated tingling sensation in her mouth and dizziness. She presented to the emergency department at her local hospital and her serum chemistries were notable for eosinophilia and elevated serum creatinine 4.32 mg/dL (baseline 1.38 mg/dL). Due to concerns of DIHS, the patient was transferred to the UCSD hospital. On presentation, the patient had a morbilliform rash on her torso and her serum chemistries were notable for an elevated absolute eosinophil count of 5000/mm^3^, elevated serum creatinine of 3.47 mg/dL and urinalysis with evidence of proteinuria but normal liver function tests. An HLA-B*58:01 test showed that the patient was positive for the risk allele (Table 1). She started on prednisone 1 mg/kg daily with a long taper and serum chemistries normalized within 1 month.

This case further illustrates the knowledge gap inherent in the current 2020 ACR guidelines that only recommend HLA-B*58:01 testing in patients of certain ethnicities. This patient, although not South East Asian or African American, carries the HLA-B*58:01 risk allele, is over the age of 60, has CKD and was prescribed allopurinol 100 mg daily. Therefore, she was at increased risk for allopurinol DIHS but based on current guidelines would not be pre-screened for the HLA-B*58:01 allele.

It is well established that ethnicity describes a person’s recent geographic ancestry and this is often used as a proxy for predicted genetics [8]. However, in genetically diverse and admixed populations such as Hispanics with varying degrees of European, Native American and African ancestry, it would be faulty to assume a person’s genetics based on their phenotypic appearance. For example, Puerto Ricans have more African and European ancestry vs. Native American ancestry compared to Mexicans who have more Native American and less African and European ancestry [8]. Therefore, it may be that Hispanics from Puerto Rico may have a higher likelihood of carrying the HLA-B*58:01 allele compared to Mexicans. Thus far, pharmacogenomic knowledge of the frequency of the HLA-B*58:01 allele in Hispanic populations is sparce and further studies are warranted.

The updated ACR guidelines are a step in the right direction and will likely be closely adhered to by practicing rheumatologists. However, allopurinol is also prescribed by primary care physicians and other specialists who may or may not follow the ACR guidelines recommendation to test for an HLA-B*58:01 allele in specific populations prior to initiating allopurinol. Therefore, we posit that a larger governing body such as the FDA would be more appropriate to provide guidance to all practicing physicians that may prescribe allopurinol. However, the US FDA, unlike the Taiwanese FDA and the Japanese FDA, has not required a warning on the allopurinol drug label alerting physicians and patients to the association between the presence of the HLA-B*58:01 allele and DIHS, a life-threatening condition [8].

We posit that in order to prevent allopurinol DIHS, the following steps must be taken. First, the US FDA should require labelling for allopurinol, alerting physicians to the association between HLA-B*58:01 and allopurinol DIHS [8]. Second, because self-identified ethnicity is not predictive of genetics, health systems and individual physicians should be justified in screening all patients for the HLA-B*58:01 allele before prescribing allopurinol [8].

Therefore, at our institution, the Department of Dermatology is working to implement a system-change in our electronic health record to alert prescribers of allopurinol to screen for the HLA-B*58:01 allele in all patients. We recently published an algorithm to help guide physicians to safely prescribe allopurinol [8]. The algorithm will be embedded into the alert which serves to educate physicians about the risk of DIHS in carriers of the HLA-B*58:01 allele but also in those with other associated risk factors, especially CKD. It has been shown that oxypurinol, an active metabolite of allopurinol, binds to the HLA-B*58:01 peptide binding groove with higher affinity and is excreted renally [5]. Therefore, patients with baseline CKD that are prescribed allopurinol, especially at doses greater than 100 mg daily, can experience DIHS secondary to oxypurinol accumulation even in the absence of HLA-B*58:01 allele [5].

## 5. Case 3: Sulfamethoxazole/Trimethoprim (Bactrim) Drug-Induced Hypersensitivity Syndrome

**Figure 2:**
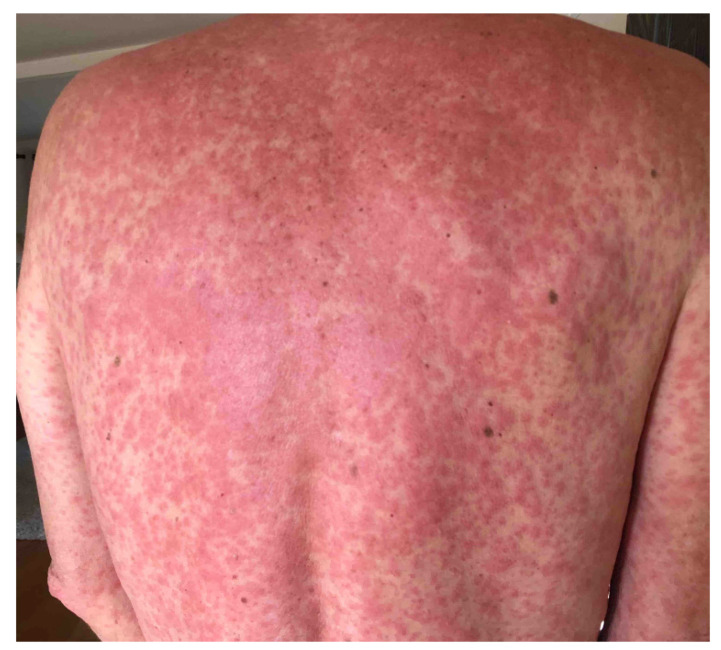
Patient with Bactrim DIHS. Morbilliform rash with 50% body surface area (BSA) involvement.

Mr. L, a 66-year-old man with a history of hypertension and a remote history of bladder cancer, presented to the Emergency Department in November 2020 with a two-day history of a morbilliform rash that began on his torso and spread to his extremities (Figure 2). His serum chemistries were notable for elevated serum creatinine 2.24 mg/dL (baseline 1.2 mg/dL), normal liver enzymes and no eosinophilia. A review of his medication history revealed that he was prescribed sulfamethoxazole/trimethoprim (Bactrim) 7 days prior for the treatment of small intestinal bacterial overgrowth. The patient denied ever having had a similar rash in the past. He denied a prodrome of viral illness such as fever, malaise, arthralgias. Virologic serologies for cytomegalovirus (CMV), Epstein–Barr virus (EBV), human herpes virus 6 (HHV6) and coronavirus 19 (COVID-19) were negative. Bactrim was discontinued and the patient was started on prednisone 1 mg/kg daily. Within two days, his serum creatinine began to normalize, his rash began to resolve and he was discharged home on a 6-week course of a prednisone taper. During his outpatient follow up with dermatology, we ordered for genetic testing for N-acetyl transferase 2 (*NAT2*) and the results showed that Mr. L harbored several *NAT2* haplotypes *5B/*6B, *5C/*6E, *5E/*12C associated with the slow acetylator phenotype (Table 2).

There is marked inter-individual and inter-ethnic variability in NAT2 acetylation. For example, 56% of US Whites are slow NAT2 acetylators compared to 44% of US Blacks [9]. This variation has been shown to be associated with an increased frequency of transitional cell bladder cancer in US Whites compared to Blacks [9]. Similar rates of NAT2 slow acetylator phenotype have been reported in other populations of European descent: 55.1% of Germans, and 65.4% of Spaniards [10]. In contrast, the NAT2 slow acetylator phenotype occurs in only 9% of Serbians [10]. Considering people of African descent, compared to 44% of US Blacks with a NAT2 slow acetylator genotype [9], 68% of Nigerians are NAT2 slow acetylators [11]. Similarly, in Asian populations, the frequency of NAT2 slow acetylator phenotype is higher in Thai populations (50%) compared to a frequency of 5–20% in other Asian populations (Japan, Korea, China) [12].

The US FDA label for Bactrim warns that hemolysis may occur in patients deficient in G6PD enzyme [13]. It also states that patients who are slow acetylators may be at higher risk for idiosyncratic adverse drug reactions. However, the label never mentions that the acetylator status can be ascertained by sequencing the *NAT2* gene [14]. Similarly, the US FDA label for other commonly used medications metabolized by NAT2 (isoniazid, procainamide and sulfasalazine) only mentions that slow acetylators may be at increased risk of adverse drug reactions but never mentions the *NAT2* gene [14]. Amifampridine, used in the treatment of Lambert–Eaton myasthenic syndrome, is the only medication for which there is a US FDA label warning of the association between NAT2 acetylator status and adverse drug reaction [13,14]. The label goes on to recommend a dose reduction of amifampridine in patients with reduced NAT2 enzyme activity [13,14].

Bactrim is widely used for the treatment of bacterial infections and prophylaxis against pneumocystis pneumonia (PCP) in immunocompromised and immunosuppressed patients [3]. In the US in 2018 alone, 8,448,278 prescriptions for Bactrim were written [15]. It is known that NAT2 slow acetylators are at increased risk of Bactrim-induced SCARs [3]. Given that 56% of US Whites, 44% of US Blacks and 5–20% of Asians are NAT2 slow acetylators [9,12], the US FDA Bactrim drug label should clearly emphasize the preventative role of *NAT2* genetic testing. In the absence of such clear guidance, clinicians and health systems cannot make adequate changes to reduce the frequency of Bactrim-induced SCARs. To address the knowledge gap of the clinical application of pharmacogenomics in the prevention of Bactrim-induced SCARs, the Department of Dermatology at UCSD is beginning to utilize pre-emptive *NAT2* genetic testing.

## 6. Case 4: Pre-emptive NAT2 Genetic Testing

Ms. D is a 72-year-old woman with a history of pemphigus vulgaris, previously treated with rituximab. She presented to the UCSD dermatology clinic in September 2020 with several weeks of mucosal and cutaneous erosions, representing a flare of her pemphigus vulgaris. A month prior, she was started on mycophenolate mofetil and prednisone. Given the extent of mucosal and skin involvement of her pemphigus vulgaris, she would likely require a long course of prednisone therapy and therefore a consideration of PCP prophylaxis with Bactrim. Prior to prescribing Bactrim, a *NAT2* genetic test was ordered and the results showed that the patient was homozygous for the NAT2*5B genotype associated with slow NAT2 acetylator status (Table 3). The risk of Bactrim-induced SCARs was discussed with the patient and she declined treatment with Bactrim opting instead for a safer alternative.

This case vignette illustrates the manner in which pre-emptive genetic testing can be performed. By performing *NAT2* genetic testing before initiating Bactrim, a careful and thoughtful discussion of the risks and benefits allowed the patient to be involved in the care of her complex disease. In so doing, we likely prevented the patient, a NAT2 slow acetylator, from developing Bactrim-induced SCAR.

To our knowledge, this is the first reported case of *NAT2* genetic testing to prevent Bactrim associated SCAR in a US dermatology clinic. Although this is not currently the standard of care in the US, it has the potential to dramatically improve patient care and reduce health care costs by preventing potentially life-threatening adverse drug reactions.

## 7. Conclusions

Although there are several examples of the utility of pharmacogenomics in preventing adverse drug reactions, in the US there is not yet the widespread adoption of pre-emptive testing [14] except for screening for the presence of the HLA-B*57:01 allele prior to prescribing abacavir which is routinely performed in the US [1]. There are many reasons for which pre-emptive pharmacogenomics is not widely practiced in the US: (1) a lack of coherent guidance from FDA drug labels; (2) the absence of clinical expertise; (3) diverse populations with many unknown pharmacogenomic markers of drug response due to being understudied; and (4) the lack of a knowledge base to facilitate clinical implementation models within health systems [14]. Presenting this case series, we demonstrated how Dermatologists and other physicians will play a role in implementing pharmacogenomics testing in the clinical setting. First, dermatologists can demonstrate how HLA-B*58:01 testing will aid in the diagnosis of allopurinol-induced SCAR by ordering the test when allopurinol is suspected as the culprit medication in a patient diagnosed with SCAR. Dermatologists and other physicians can also advocate for changes within health systems toward implementing pre-emptive HLA-B*58:01 testing prior to prescribing allopurinol to prevent future SCAR. The third case demonstrates how the NAT2 slow acetylator phenotype predisposes to Bactrim-induced SCAR. In the fourth case, we show how physicians can implement pre-emptive *NAT2* genetic testing within their own practice to prevent Bactrim-induced SCAR. This serves as an example of the utility of *NAT2* genetic testing to prevent Bactrim-induced SCAR for prescribing physicians of any specialty. In our experience, insurance companies have covered the cost of testing because it was associated with clinical care.

In the broader context, this case series serves as a template for how to advocate for more widespread pharmacogenetic testing to prevent SCAR induced by other culprit medications such as lamotrigine, carbamazepine and phenytoin and the associated risk alleles HLA-B*15:02 and HLA-A*31:01.

## Figures and Tables

**Table 1:** Case series of allopurinol DIHS. M—male, F—female.

Sex	Age (Years)	Race/Ethnicity (Self-Identified)	HLA-B*58:01	Allopurinol Dose (Daily)	Baseline Serum Creatinine (mg/dL)
M	42	Black	Positive	300 mg	2.06
F	81	Hispanic	Positive	100 mg	1.38

**Table 2:** Case of Bactrim DIHS. M—male.

Sex	Age (Years)	Race/Ethnicity (Self-Identified)	*NAT2* Genotype	Medication	Baseline Serum Creatinine (mg/dL)
M	66	White	*5B/*6B, *5C/*6E, *5E/*12C	Bactrim	1.2

**Table 3:** Pre-emptive *NAT2* genetic testing prior to prescribing Bactrim. F—female.

Sex	Age (Years)	Race/Ethnicity (Self-Identified)	*NAT2* Genotype	Medication	Baseline Serum Creatinine (mg/dL)
F	72	White	*5B/*5B	Bactrim	0.66

## Data Availability

Data is contained within the article.

## References

[1] Chang C.-J., Chen C.-B., Hung S.-I., Ji C., Chung W. (2020). Pharmacogenetic Testing for Prevention of Severe Cutaneous Adverse Drug Reactions. Front. Pharmacol..

[2] Yu K.-H., Yu C.-Y., Fang Y.-F. (2017). Diagnostic utility of HLA-B*5801 screening in severe allopurinol hypersensitivity syndrome: An updated systematic review and meta-analysis. Int. J. Rheum. Dis..

[3] Sim E., Abuhammad A., Ryan A. (2014). Arylamine N-acetyltransferases: From drug metabolism and pharmacogenetics to drug discovery. Br. J. Pharmacol..

[4] Wang D., Para M.F., Koletar S.L., Sadee W. (2011). Human N-acetyltransferase 1 (NAT1) *10 and *11 alleles increase protein expression via distinct mechanisms and associate with sulfamethoxazole-induced hypersensitivity. Pharm. Genom..

[5] Keller S.F., Lu N., Blumenthal K.G., Rai S.K., Yokose C., Choi J.W.J., Kim S.C., Zhang Y., Choi H.K. (2018). Racial/ethnic variation and risk factors for allopurinol-associated severe cutaneous adverse reactions: A cohort study. Ann. Rheum. Dis..

[6] Jutkowitz E., Dubreuil M., Lu N., Kuntz K.M., Choi H.K. (2017). The cost-effectiveness of HLA-B*5801 screening to guide initial urate-lowering therapy for gout in the united states. Semin. Arthritis Rheum..

[7] FitzGerald J.D., Dalbeth N., Mikuls T.R., Brignardello-Petersen R., Guyatt G., Abeles A.M., Gelber A.C., Harrold L.R., Khanna D., King C. (2020). 2020 American College of Rheumatology Guideline for the Management of Gout. Arthritis Rheumatol..

[8] Ikediobi O. (2020). A Personalized Medicine Approach to Guide Allopurinol Use and Prevent Serious Adverse Events: Ethnicity Is Not a Proxy for Genetics. Clin. Pharmacol. Ther..

[9] Muscat J.E., Pittman B., Kleinman W., Lazarus P., Stellman S.D., Richie J.P. (2008). Comparison of CYP1A2 and NAT2 phenotypes between black and white smokers. Biochem. Pharmacol..

[10] Djordjevic N., Carrillo J.A., Ueda N., Gervasini G., Fukasawa T., Suda A., Jankovic S., Aklillu E. (2011). N-acetyltransferase-2 (NAT2) gene polymorphisms and enzyme activity in Serbs: Unprecedented high prevalence of rapid acetylators in a white population. J. Clin. Pharmacol..

[11] Kotila O.A., Fawole O.I., Olopade O.I., Ayede A.I., Falusi A.G., Babalola C.P. (2019). N-acetyltransferase 2 enzyme genotype–phenotype discordances in both HIV-negative and HIV-positive Nigerians. Pharm. Genom..

[12] Sabbagh A., Langaney A., Darlu P., Gérard N., Krishnamoorthy R., Poloni E.S. (2008). Worldwide distribution of NAT2 diversity: Implications for NAT2 evolutionary history. BMC Genet..

[13] Food and Drug Administration Table of Pharmacogenomic Biomarkers in Drug Labeling. http://www.fda.gov.

[14] Whirl-Carrillo M., McDonagh E.M., Hebert J.M., Gong L., Sangkuhl K., Thorn C.F., Altman R.B., Klein T.E. (2012). Pharmacogenomics Knowledge for Personalized Medicine. Clin. Pharmacol. Ther..

[15] U.S. Outpatient Drug Usage Statistics ClinCalc DrugStats Database Version 21.1. Prescription Data Source: Medical Expenditure Panel Survey (MEPS) 2008–2018. www.clincalc.com/DrugStats/.

